# Effect of Educational Intervention Based on Self-Efficacy Theory on the Caring Behaviour of Mothers Who Have Children With Cancer

**DOI:** 10.34763/jmotherandchild.20232701.d-22-00065

**Published:** 2023-08-31

**Authors:** Maryam Barani, Laleh Hassani, Amin Ghanbarnejad, Mohammad Ali Molavi

**Affiliations:** Department of Pediatrics, Pediatrics Hospital, Hormozgan University of Medical Sciences, Bandar Abbas, Iran; Mother and Child Welfare Research Center, Hormozgan University of Medical Sciences, Bandar Abbas, Iran; Department of Public Health, School of Health, Social Determinants in Health Promotion Research Center, Research Institute for Health, Hormozgan University of Medical Sciences, Bandar Abbas, Iran; Department of Pediatrics, School of Medicine Pediatrics Hospital, Hormozgan University of Medical Sciences, Bandar Abbas, Iran

**Keywords:** self-efficacy theory, educational intervention, caring behaviour, children with cancer

## Abstract

Self-efficacy, as one of the concepts of the empowerment model, plays a role in increasing caring behaviour. Accordingly, our study aimed to evaluate the effect of educational intervention based on self-efficacy theory on the caring behaviour of mothers who have children with cancer. This quasi-experimental study was conducted on all mothers (N = 86) of children with cancer undergoing chemotherapy who were referred to Bandar Abbas Children's Hospital. All mothers participated in 10 training sessions based on the four foundations of self-efficacy theory. The results indicated a significant increase in the mean score of mothers’ self-efficacy at first and second follow-ups respectively by 10.2 and 10.9 after intervention (P < 0.001). Furthermore, the mean score of caring behaviour was increased after the intervention for the first and second follow ups by 24.6 and 25.9 from the baseline respectively (P < 0.001). The results of the present study indicated that an educational program for mothers increased their caring behaviour and self-efficacy with the increase of self-efficacy, mothers’ caring behaviour was promoted. Authorities and practitioners are suggested to pay more attention to designing educational programs based on health models and theories.

## Introduction

Since children with cancer receive a lot of home treatment, parents feel more responsibility for issues including drug use control, poison control, drug side effects, and contact with the treatment team [1]. Parents, particularly mothers, as the most significant people in a child's support system, are often unaware of the causes of the disease and the ways in which they are treated, the economic consequences of the child's disease, and the frequent hospitalization of the child, which can lead to stress and psychological problems, as well as adverse effects on the child and the treatment process [2]. Thus, family members must reorganise their roles and interactive patterns regarding inside and outside relationships, and work to adapt to the new situation [3]. Thus, the performance of the whole family is influential [4]. Increased psychological pressure on caregivers can have consequences including family isolation, loss of hope for social support, disruption of family relationships, inadequate patient care, and, ultimately, abandonment of the patient [5]. Since the past two decades have witnessed a shift in healthcare from hospital to at-home care, more than 90% of outpatient and inpatient cancer treatments are now available at home. One of the factors that may facilitate this transition from hospital to home is proper education of the patient and his/her family members and other caregivers, as well as their active involvement in providing care. In addition, empowerment is one of the critical actions of nursing to attract participation and education of patients and their caregivers [6]. Empowerment, as a participatory educational approach, requires looking at the family and its needs as the core of care. One of the concepts of the empowerment model is self-efficacy [7]. The concept of self-efficacy was first introduced by Bandura in 1993 as a probable measure of one's trust in a particular behavioural competence or a chain of specific behaviours intended to control and manage perceived situations [8]. The ability to exert control over the intellectual/motivational processes present in one's behaviour is one of the distinctive features of human behaviour [9]. Self-efficacy has been defined as a mediating mechanism affecting the underlying motivational, emotional, and selective processes. Perceived self-efficacy is not related to the number of skills but to what one believes and can do under certain circumstances. Skills are easily influenced by one's doubts. Effective performance requires skill coupled with self-efficacy [10]. Research has indicated that self-efficacy can predict one's performance and change it as a result of learning [11], experience, and feedback. At the same time, many factors like personal knowledge, physical status, self-esteem, interpersonal environment, time available, complexity of tasks, and stress can affect self-efficacy and behavioural outcomes [12,13]. According to the results of various studies, the rate of depression in mothers with children with cancer is more than 80% [14,15]. In addition, the depression of parents, especially mothers, had an inverse effect on their self-efficacy, so that mothers with high degrees of depression had low self-efficacy. Eventually, this leads mothers’ performance as the main caregiver of children with various cancers to decrease [16,17]. In different studies, the results show that the status of self-efficacy in the mother remains average [18,19,20,21]. Interventions based on the Theory of Self-Efficacy (TSE) have demonstrated the effectiveness of this theory in improving or acquiring skills. TSE has been used in numerous studies like women's general health status [22], physical activity [23], adopting AIDS prevention behaviour [24], increasing women's awareness in preventing violence against women [25], maternal competence in women [26], and self-efficacy of mothers of children with autism [27,28]. Because of the nature of cancer and its psychological effects on mothers as caregivers, our study aimed to evaluate the effect of maternal education on how to care for a child with cancer, and their skill development, based on the TSE.

## Material and method

### Study design and participants

This research study was conducted between October 2019 and February 2021 in Bandar Abbas, Iran. This study was quasi-experimental. The research hypothesis was the effect of intervention based on TSE on the mothers’ caring behaviours. The participants (*n* = 86) were all mothers of children with cancer undergoing chemotherapy who had been referred to Bandar Abbas Children's Hospital. Inclusion criteria were being the mother of a child, having at least one child undergoing chemotherapy, and giving consent to participate in the study. Exclusion criteria were non-attendance at educational sessions (absence of more than two sessions), deterioration of the child's health situation, and failure to complete the questionnaire.

### Sampling

The children's hospital, which is the only referral hospital in the cities of Hormozgan Province, provides short-term and outpatient services for children with various cancers. The maximum number of children admitted with cancer per month was not greater than 100. Therefore, all mothers of children who were referred for treatment were included in the study. Due to the time interval that existed between the first stage of questioning and the intervention, and the result of the intervention and follow-up, some people died between stages and were not present during the post-intervention test. Some were excluded after moving from Bandar Abbas to another city, where it was not possible to follow up with them. 86 participants were considered through all stages.

### Data collection tool

The required data were collected by Sherer's questionnaire for measuring the mothers’ self-efficacy. There was also a researcher-made questionnaire used for measuring maternal caring behaviour. The demographic questions included mother's age, child's age, marital status, mother's education, mother's occupation, household size, number of chemotherapy sessions, year of diagnosis, and whether mothers had multiple children. The researcher-made questionnaire was designed with 30 questions to assess the parental care of children with cancer in the field of nutrition, side effects of chemotherapy, and how to serve food and manage gastrointestinal disorders caused by chemotherapy and changes in appearance, especially hair loss. In this research, to determine the Content Validity Index (CVI) and the Content Validity Ratio (CVR), a panel of experts (*n* = 10) reviewed scale items. The CVI and CVR scores of the scale were 0.876 and 0.892, respectively.

The questionnaire had a minimum of 30 and a maximum of 150 points. The situation during and after chemotherapy was measured on a 5-point Likert scale (where 5: I can, 4: I’m sure, 4: I try to, 2: I’m not sure, and 1: I cannot). In order to get the most appropriate answer from the parents, we observed the expression of their pupils, so that if the parents did not understand the options, they could respond via their pupils according to their satisfaction with the way of care [29]. The panel of experts, who had different specialties including education and health promotion, epidemiology, biostatistics, and blood and cancer specializations in children, was used to determine the validity of the questionnaire. The questionnaires were distributed randomly to determine the reliability of the study population. The researcher-made questionnaire was then filled out two times in a 10-day interval. There was no significant difference between the two times (P > 0.05). Cronbach's alpha was used for determining the internal reliability of the questionnaire, which was found to be α = 0.87. It is to be noted that due to the small sample size, none of the participants in the pilot study was excluded from the whole study. The Sherer's self-efficacy questionnaire, whose validity and reliability have been confirmed previously in Iran [30], included 17 items on a 5-point Likert scale (where 1: strongly disagree, 2: disagree, 3: intermediate, 4: agree, and 5: strongly agree). Six of the 17 questions did not need to be changed, and the rest of the questions were reversed and/or changed.

### Intervention

Both questionnaires were completed by the researcher, using verbal information for those parents who were illiterate. After collecting the first stage questionnaires and analysing the data, educational content was designed based on the results of the data analysis. An educational program, including a set of caring behaviour sessions in groups of 8–9 participants (each session lasting 45–60 min) was implemented. Ten interventional education sessions, divided into 2 or 3 sessions each (focusing on performance outcomes, vicarious experiences, verbal persuasion, and physiological feedback), were held within one month. The training took place in the hospital. The educational program included lectures with questions and answer sessions, group discussions, and educational clip broadcasts. In addition, an instructive booklet was provided for mothers at the end of the intervention period. Educational interventions for the mothers’ caring behaviours for children with cancer were based on TSE, including performance outcomes, vicarious experiences, verbal persuasion, physiological feedback, and content production, according to the books on childcare for cancer. The training used the infrastructure of TSE in addition to the content produced. Nurses also attended the training sessions to provide the parents with hospitalization and discharge training. In order to investigate the continuity of caring behaviour in the mothers with children with cancer, the study was designed with two follow-up stages. The first stage was evaluated three months post-intervention and the second follow-up was evaluated six months post-intervention.

### Data analysis

SPSS software (ver. 25) was utilised for data analysis. The normal distribution of quantitative data was examined using the Kolmogorov-Smirnov test, indicating the normal distribution of all data. Descriptive statistics were reported as mean ± SD for quantitative variables and as frequency (%) for categorical variables. Repeated Measures ANOVA and post hoc with Bonferroni adjustment were used. A linear mixed model was performed to assess the effect of time and self-efficacy on the caring behaviour adjusting for family size, mother's age, mother's job, mother's education, and frequency of chemotherapy sessions. The significance level was set at *P* < 0.05.

## Results

In this study, 86 mothers who had children with cancer were studied. 83.8% of the mothers were between 20 and 40 years old. 55.8% of children were 5 years old or younger. Other demographic data of the study population are provided in Table 1.

**Table 1.** Demographic characteristics of mothers caring for children with cancer and undergoing chemotherapy

**Variable**	**No. (%)**	**Variable**	**No. (%)**
Mother's age		Marital Status	
(20–30) year	36 (41.9)	Married	81 (94.2)
(31–40) year	36 (41.9)	Divorced	2 (2.3)
>41 years	14 (16.2)	Widow	3 (3.5)
Occupation		Child's age	
Homemaker	78 (90.7)	0–5 years	48 (55.8)
Employed	8 (9.3)	>5 years	38 (44.2)
Level of education		Number of chemotherapy sessions per month	
Illiterate	16 (18.6)	Less than 10 sessions	27 (31.4)
Under diploma	36 (41.8)	(10–20) sessions	19 (22.1)
Diploma / University	34 (39.6)	More than 20 sessions	40 (46.5)

**Figure 1.**
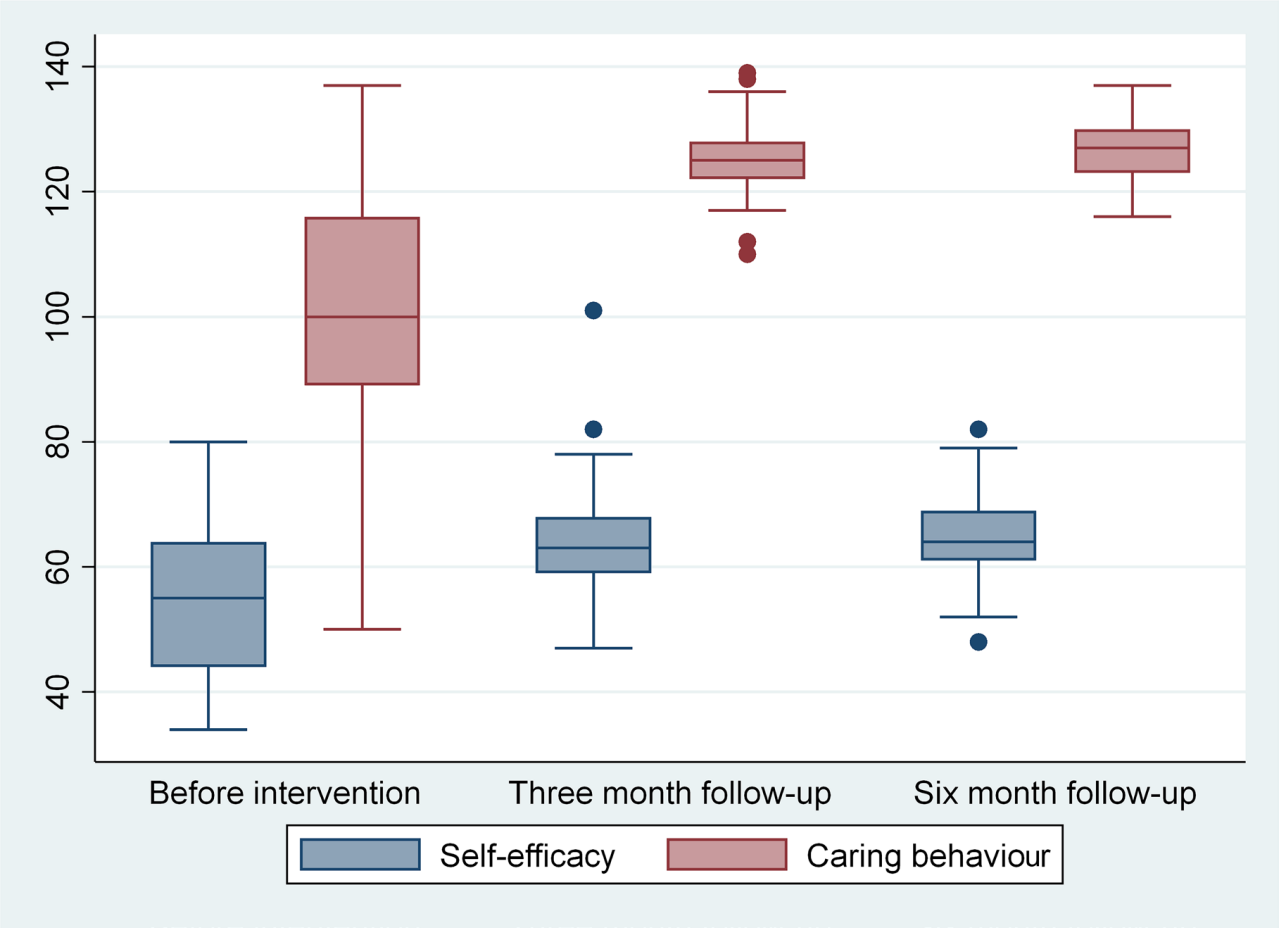
Boxplot and distribution of caring behaviour and self-efficacy scores across the evaluation period.

The results showed that the M±SD of mothers’ self-efficacy was 53.63±11.41. After intervention, the mean self-efficacy score was 63.83 in the first follow-up and 65.56 in the second follow-up. The mean score of caring behaviour before intervention was 100.73±18.91, which increased to 125.4 in the first follow-up and 126.63 in the second follow-up post-intervention. Distribution indices for self-efficacy and mothers’ caring behaviour before intervention and in the first and second follow-ups are displayed here as a boxplot (Figure 1).

The repeated measure ANOVA test was used to investigate the effect of follow-up times on changes in mothers’ self-efficacy and caring behaviour. The results showed that the change in self-efficacy score and care behaviour in the different follow-up stages was statistically significant (*P* < 0.001). Therefore, it can be said that educational intervention changed the level of mothers’ self-efficacy and caring behaviour.

As shown in Table 2, the score of self-efficacy in the first and second follow-ups increased significantly (*P* < 0.001) in comparison with the pre-test. There was also no significant difference between the first and second follow-ups (*P* = 0.287). Furthermore, the score of caring behaviour in the first and second follow-ups increased significantly (*P* < 0.001) in comparison with the pre-test. However, there was no significant difference between the first and second follow-ups (*P* < 0.001).

**Table 2.** Comparison of the mean scores of mothers’ self-efficacy and caring behaviour in three stages

**Variable**	**Time of study**	**Means of differences**	**Confidence interval 95% for means**		**P-value**

			**Low limit**	**High limit**	
Self-efficacy	Pre-test vs. first follow-up	10.2	6.5	13.9	>0.001
	Pre-test vs. second follow-up	10.9	7.5	14.3	>0.001
	First follow-up vs. second follow-up	0.73	−0.33	1.8	=0.287
Caring behaviour	Pre-test vs. first follow-up	24.6	19.6	29.7	>0.001
	Pre-test vs. second follow-up	25.9	20.8	31.0	>0.001
	First follow-up vs. second follow-up	1.29	0.60	1.98	>0.001

The Linear Mixed Model was used to investigate the relationship between self-efficacy and caring behaviour and the effect of intervention on their changes over time by adjusting the effect of family size, mother's age, mother's occupation, mother's education, and number of chemotherapy sessions. The results showed that caring behaviour was not related to the mothers’ age, occupation, or education. Increase of household size was inversely related to caring behaviour. Self-efficacy and number of chemotherapy sessions were also directly related to caring behaviour (Table 3). Also, based on the results of the Linear Mixed Model, the effect of time was significant; therefore, it can be claimed that there was a significant change in caring behaviour score in the first and second follow-ups in comparison with the baseline before intervention.

**Table 3.** Parameter estimate of the Linear Mixed Model

**Parameter**	**Estimate**	**Std. Error**	**P-value**
Intercept	75.24	7.44	<0.001
Time: Baseline^a^	-	-	<0.001
Time: First follow-up	20.04	1.77	
Time: Second follow-up	20.99	1.79	
Self-efficacy	0.45	0.08	<0.001
Family size: 2 persons^a^	-	-	0.017
Family size: 3 to 4 persons	−5.14	4.88	
Family size ≥5 persons	−10.68	5.20	
Mother's age	0.20	0.12	0.114
Job: Housewife^a^	-	-	0.95
Job: Employed	−0.17	2.66	
Education: Illiterate^a^	-	-	0.25
Education: Under diploma	3.08	2.05	
Education: Diploma or university degree	1.22	2.65	
Chemotherapy: under 10 sessions^a^	-	-	0.027
Chemotherapy: 10 to 20 sessions	3.99	2.09	
Chemotherapy: >20 sessions	4.63	1.77	

## Discussion

This study aimed to determine the effect of mothers’ education and skill development according to TSE on their caring behaviour for a child with cancer and the continuation of care in Bandar Abbas Paediatric Hospital, which is the only paediatric cancer centre in the province. This study used innovations in the methodology and application of TSE to enhance the caregiving power of mothers’ caring behaviour for children with cancer in Iran.

The results indicated that there was a significant difference in the mean score of self-efficacy after the intervention, because parents need to be more sensitive to their children's needs and emotions and pay more attention to their children. These abilities of parents — to provide adequate nutrition for their children, provide preventive and corrective health care, diagnose the signs and symptoms of illness in children, develop habits to maintain cleanliness, and encourage children to get proper rest time and outdoor activities for their children — are all things that should be considered in children with cancer because of their special needs. This finding is in line with the results of other studies [31,32,33]. Studies aimed at investigating the effect of family-centred education on increasing parental awareness and self-efficacy in weight control and physical activity, and caring for a child with asthma and epilepsy, also showed that education can increase self-efficacy and improve home care effectively [34,35,36,37]. Self-efficacy is a principle that links knowledge and behaviour. For this reason, a sense of self-efficacy enables individuals to do extraordinary work using skills to overcome obstacles. Performance requires both skill and belief in the ability to perform that skill. Self-efficacy is a pre-requisite to a behaviour; so special attention should be paid to increasing self-efficacy [36]. In the present study, considering that the follow-up was done in two stages with intervals of 3 and 6 months, the mean scores of self-efficacy in the first and second stage follow-up had no significant differences, which indicates the stability of self-efficacy [38]. Consistent with the results of a clinical trial conducted on the continuation of mothers’ breastfeeding self-efficacy, our findings revealed that the mean scores of self-efficacy after educational intervention increased at two follow-up intervals [39]. In contrast, another study aiming to examine the impact of parental education on the self-efficacy of mothers of children with autism showed that mothers’ education did not significantly increase their self-efficacy in the first and second follow-ups. The results further indicated that parental education and nurturing skills were not effective in enhancing parental self-efficacy [40]. This difference can be due to lack of attention to emotional or marital problems of parents, the lack of social support during the implementation of the program, and the compactness of the treatment and content sessions, as well as the questions that examine the general feeling of parental self-efficacy rather than measuring parental self-efficacy in managing their children's problems.

The difference between the mean scores of caring behaviour before and after the intervention in the two stages shows the effect of educational intervention to improve mothers’ caring behaviour, which is consistent with the results of other studies [41,42,43]. Another study on the effect of education of mothers of children with cancer undergoing chemotherapy on prevention of the gastrointestinal side effects showed that educational intervention had a significant impact on reducing these effects [6,44]. An investigation on the effect of educational intervention on the home care behaviour of parents who had children with cancer undergoing chemotherapy indicated that the intervention was effective in reducing the effects of chemotherapy [45]. In our study, the parents received education about caring behaviours including how to reduce the side effects of chemotherapy at home. The results showed that the educational intervention had an effect on parental caring behaviours, which included reducing the effects of chemotherapy. The second stage findings revealed that parental caring behaviour, in addition to being persistent, also increased over time, probably due to the experiences they gained and used during this period. In a study performed on parents of children with diarrhoea, vomiting and pneumonia, the effect of training on parents’ performance and satisfaction showed that the training program had a significant effect on parental care compared to the control group [46]. Furthermore, in a study designed to empower parents in caring for a child with leukaemia, the results indicated that the intervention could be effective in parental satisfaction, especially in the mother, regarding how to care for a child with leukaemia and decrease the side effects of chemotherapy [47]. The result of a study on caregivers of people undergoing bypass surgery also showed that the intervention led to increased self-efficacy and improved care behaviour, and thus, increased care for patients undergoing surgery [48].

Our results showed that with increasing the duration of the study, and even after the intervention at the time of follow-up, the mothers’ caring behaviour was still significantly related to self-efficacy. In other words, mothers are aware of their role in caring for their children and do consider this ability as intermittent. They are able to maintain the continuity of behaviour by maintaining and promoting self-efficacy in their caring behaviour. Bandura argued that people with high self-efficacy are more likely to face challenges that need to be controlled and threats that need to be avoided. Given this confidence, the ability to increase their ability to cope with a variety of challenges will be relatively easier. Thus, as a hypothesis, this sense of confidence for success in solving the challenge is associated with lower levels of negative emotional reactions both before and after intervention [49,50,51]. The Linear Mixed Model results showed that an increase in family size was inversely related with mothers’ caring behaviour. In fact, in families of smaller sizes, the mother's caring behaviour was better, and the relationship became more meaningful. In other words, in larger families, the mother could not be more focused on caring for her child and providing services. This would reduce the mother's caring behaviour. In addition, she would not be able to benefit from various trainings to perform caring behaviour and overcome obstacles [52,53]. The number of chemotherapy sessions was also effective in predicting performance of caring behaviour. There is also the fact that caring behaviour is very important in treatment, and chemotherapy is done to reduce the invasion of cancer cells to other tissues. This can be achieved by completing the course of chemotherapy. The data analysis results also confirmed such a relationship. On the other hand, a higher number of chemotherapy sessions leading to higher caring scores can be attributed to an increased severity of the disease, and the fact that the mother would be able to accompany the child to perform chemotherapy with more ability due to understanding the severity of the disease [54,55].

This study had some limitations, including a small sample size that may influence the attendance of all children with a single treatment centre, as well as the death of patients or discontinuation of treatment, and the difficulty of working with parents with special psychological conditions (including caring for a child with cancer, problems with parenting, and attending classes), as well as having too many questions and distributing questionnaires, and not having a control group to better evaluate intervention.

## Conclusion

The results of this study indicated that the theory-based educational program for mothers improves their caring behaviour and self-efficacy. As education is one of the main pillars of health care, it seems that authorities and practitioners should pay more attention to designing.

### Key messages

- Mothers’ self-efficacy is initially moderate, and can rise over time.
- Interventional approaches based on theory are more effective.
- The results of our study showed that mothers’ self-efficacy is effective to improve their caring behaviour.
- Nurses and physicians can also motivationally encourage mothers to increase their self-efficacy and improve their care performance.
- Mothers’ caring behaviour can be improved by educating them while treating their sick child.
